# The Use of Epoetin-**α** in Revision Knee Arthroplasty

**DOI:** 10.1155/2012/595027

**Published:** 2012-07-01

**Authors:** Lawrence A. Delasotta, Ashwin V. Rangavajjula, Michael L. Frank, Jamie L. Blair, Fabio R. Orozco, Alvin C. Ong

**Affiliations:** ^1^Department of Surgery, Temple University, Philadelphia, PA 19140, USA; ^2^Thomas Jefferson University, Philadelphia, PA 19129, USA; ^3^The Richard Stockton College of New Jersey, Pomona, NJ 08240, USA; ^4^Rothman Institute, Egg Harbor Township, NJ 08234, USA; ^5^Thomas Jefferson University Hospital, Philadelphia, PA 19129, USA; ^6^AtlantiCare Regional Medical Center, Egg Harbor Township, NJ 08234, USA

## Abstract

*Introduction*. To evaluate the efficacy of epoetin-**α** prior to revision total knee arthroplasty, we hypothesized that epoetin-**α** will reduce blood transfusion. *Methods*. Eighty-one patients were compared in this retrospective review; twenty-eight patients received our dosing regimen. All patients were mildly anemic. Epoetin-**α** to control (1 : 2) patient matching occurred so that one of two attending surgeons, gender, BMI, complexity of surgery, ASA score, and age were similar between groups. The clinical triggers for blood transfusion during or after the procedure were determined based on peri- and postoperative hemoglobin levels, ASA score, and/or clinical symptoms consistent with anemia. Blood salvage was not used. *Results*. Blood transfusion and length of stay were lower in the study group. None of the patients who received epoetin-**α** underwent transfusion. Hemoglobin increased from 11.97 to 13.8, preoperatively. Hemoglobin at day of surgery and time of discharge were higher. Gender, BMI, ASA score, total and hidden blood losses, calculated blood loss, preop PLT, PT, PTT, and INR were similar between groups. One Epogen patient had an uncomplicated DVT (3.6%). *Conclusions*. Epoetin-**α** may have a role in the mildly anemic revision knee patient. It may also decrease patient length of stay allowing for earlier readiness to resume normal activities and/or meet short-term milestones. A randomized study to evaluate the direct and indirect costs of such a treatment methodology in the mildly anemic revision patient may be warranted.

## 1. Introduction

Revision knee arthroplasty (TKA) is known to cause substantial blood loss. It also increases blood transfusions throughout the perioperative period [1]. Losses can typically range from 1 to 1.5 L (~3.85 ± 1.4 g hgb) [2–4]. Furthermore, revisions may require as much as 3 to 4 units of transfused blood [1]. Since substantial blood loss increases the need for transfusion and may extend length of stay, we are interested in the effect of preoperative epoetin-*α* on the revision knee patient. 

Allogeneic transfusion is associated with numerous risks [1, 5–7]. Preoperative autologous donation can be inconvenient, wasteful, and expensive [8]. It can also induce anemia and therefore should be avoided in the mildly anemic patient (preoperative hemoglobin (Hb) between 10.0 g/dL and 13.0 g/dL) [8]. However, recent literature suggests knee arthroplasty patients may benefit from pre-operative epoetin-*α* injection [9]; it's use in cancer, chronic renal failure, critical care, and orthopaedic trauma patients has also shown promising results [10].

A paucity of information is available on preoperative epoetin-*α* use in the revision knee patient. We studied a consecutive series of mildly anemic patients (10.0–13.0 g/dL) who underwent a revision knee surgery. We hypothesized that mildly anemic patients who receive epoetin-*α* will receive fewer transfusions. This patient population is thought to have a fourfold and fifteenfold transfusion rate increase over those with hemoglobin levels between 13.0–15.0 g/dL and >15 g/dL, respectively [11, 12].

## 2. Methods

Following Institutional Review Board (IRB) approval, we performed this retrospective analysis. Between January 2007 and May 2010 there were 81 patients who met our inclusion and exclusion criteria (see below); twenty-eight of these patients electively received epoetin-*α*. All patients received a revision knee surgery for prosthesis wear out and/or loosening. All surgical procedures were elective. The following cases were excluded from the study: subjects with pre-operative Hb values less than 10 g/dL or greater than 13 g/dL, patients with hematological diseases or coagulation disorders, a history of prior deep venous thrombosis or a pulmonary embolus, periprosthetic infection, and subjects who received a postoperative drain. We defined mildly anemic patients as those with an Hb level at or below 13 g/dL and at or above 10 g/dL [11, 12]. Any patient, during pre-operative evaluation, who had an Hb level of <9 g/dL, was referred to a hematologist for further evaluation. 

When a hemoglobin level was ≥10 and ≤13 g/dL, then epoetin-*α* was considered 21, 14, and 7 days prior to surgery. All mildly anemic patients at our site of practice are referred to an expert who explains the risks and benefits of treatments for preoperative anemia—all risks associated with epoetin-*α* use were discussed. Patients that did and did not receive epoetin-*α* who met the study inclusion criteria were patient-matched (1 : 2) according to one of two attending surgeons, gender, bmi, complexity of surgery, ASA score, and age. All patients were offered oral multivitamins, vitamin B12, folic acid, and iron.

The preoperative workup, surgical technique, anesthesia, and postoperative management of patients in both groups were identical. All surgeries were completed under combined spinal-epidural anesthesia, with tourniquet control. A straight medial parapatellar approach was made to the left or right knee incorporating the old incision. A synovectomy occurred in all cases. All knees were cemented. We do not use a lateral release. Neither cell saver nor drains were used; at our institution, it is not a routine practice. Through 4-weeks post-op, proper anticoagulant (either oral warfarin or subcutaneous enoxaparin) was administered to the patient. The target INR for all patients was 2.0–2.5. The first dose of prophylactic antibiotic was administered within one hour prior to incision and then continued for the first 24 hours. The clinical triggers for blood transfusion during or after the procedure were determined based on peri- and postoperative hemoglobin levels, the ASA score (American Society of Anesthesiologists) of the patient, and/or clinical symptoms consistent with an anemic picture.

We used a chi-square test for testing the proportions of cases receiving blood, and Student's *t*-test and chi-square were used for comparing the continuous and categorical variables, respectively. For the statistical analysis, version 18 of PASW Statistics (SPSS Inc., an IBM Company Headquarters, Chicago, IL) was used. A *P* < 0.05 was considered statistically significant.

## 3. Results

A total of 81 mildly anemic patients who met our study criteria had revision knee surgery from January 2007 to May 2010. The records of these 81 patients were reviewed and no differences in demographic data between cohorts for age (64.4 versus 63.7 yrs), gender (75% versus 75%), or BMI (32.5 versus 35.9 kg/m^2^) were found. The following patient blood values were similar: preoperative PT (11.0 versus 10.5), preoperative PTT (32.7 versus 29.2), preoperative INR (1.06 versus 1.056), and preoperative platelet count (237,360 versus 263,070 per mm^3^) (*P* > 0.05) (Table 1). 

The distribution of patients between cohorts revealed no difference in ASA score (*P* = 1.00). The median postoperative total blood loss was 1778 mL for controls and 1555 mL for cases. The median hidden blood loss was 1682 mL for controls and 1397 mL for cases. There was no difference in average total blood loss between groups (1737 versus 1670) (*P* = 0.725). There was no difference in average hidden blood loss (1643 versus 1578) (*P* = 0.7459). The calculated blood loss (CBL) between cohorts was not different (1401 mL versus 1668 mL (*P* = 0.140)) (Table 2) [13, 14]. 

At admission, the mean hemoglobin level was higher in the epoetin-*α* group (13.8 g/dL versus 12.2 g/dL) (*P* = 0.001); the discharge hemoglobin level was higher in the epoetin-*α* group (10.6 g/dL versus 9.69 g/dL) (*P* < 0.001). There was no difference in mean duration of surgery between cohorts (epoetin-*α* versus control: 85.4 minutes versus 92.66 minutes) (*P* = 0.276) (Table 2). One of the patients in the control group developed cellulitis four days after surgery which was completely resolved with antibiotic treatment. Another control patient developed a pulmonary embolus three days after the indexed operation that was treated with an inferior vena cava filter. A patient developed a hemarthrosis five days post-op and another patient developed a mechanical failure. One patient in the epoetin-*α* group (65-year-old male) passed away seven months after the index procedure due to cardiac arrest. An additional patient developed an uncomplicated acute postoperative deep venous thrombosis. No other minor or major complication, either local or systemic, was recorded.

In the epoetin-*α* group, 0% of the patients (0 of 28) required blood transfusion. This was significantly lower than the control group where 53% of the patients (28 of 53) received at least one unit of blood transfusion (*P* = 0.0001). The purpose of epoetin-*α* use preoperatively is to optimize the level of hemoglobin prior to surgery; for our patients who received an initial complete blood count at our institution (*n* = 14 of 28), the hemoglobin increased from 11.97 g/dL to 13.8 g/dL preoperatively (*P* = 0.001). The average quantities of epoetin-*α* doses received were 2.86. The epoetin-*α* cohort had a shorter hospital stay (3 versus 3.67, *P* = 0.042) (Table 2).

The index revision surgery was defined as any procedure in which at least tibial, patellar, femoral, or polyethylene components were exchanged. In the epoetin-*α* cohort, 23 (82.1%) patients had a tibia and femur revision; the remaining 5 (17.9%) received a single-component revision, 2 (7.10%) were polyethylene exchanges and 1 was for a patella (3.60%), femur (3.60%), and tibia (3.60%), respectively. In the control cohort, 43 (84.3%) patients had a tibia and femur revision; the remaining 10 (17.5%) received a single-component revision, 4 (7.01%) were polyethylene exchanges, 3 (5.30%) were patella, 2 (3.51%) tibial, and 1 (1.75%) femoral (Table 3).

## 4. Discussion

While preoperative hemoglobin levels are a known risk factor for peri- and postoperative transfusion needs in knee arthroplasty [15–17], this is the first study to assess the effect of preoperative epoetin-*α* use on the mildly anemic revision knee patient. We report that our patients did not require transfusion, had higher postoperative and discharge hemoglobin levels, and had a shorter length of stay. Epoetin-*α* successfully increased the preoperative hemoglobin levels (from 11.97 g/dL to 13.93 g/dL, resp.). One patient had a deep venous thrombosis (3.6%), postoperatively. 

While epoetin-*α* may be costly, its use in selected patient groups may actually reduce total cost (direct and indirect) during knee arthroplasty [18, 19]; however, Moonen et al. [9] reported that epoetin-*α* injection, supplemented by ferrofumerate tablets, increased the direct cost per patient when compared with a retransfusion system. They did note that without the collection of indirect costs a true cost-effectiveness analysis could not be performed. Although the retrospective nature of our study prevented a worthwhile cost analysis, our experience working with epoetin-*α* in the mildly anemic patient has been safe and efficacious, and we hypothesize that the elevated postoperative hemoglobin level may increase the patient's short-term outcome [20]. A Future randomized study should include direct and indirect cost data as part of their analysis. 

Cushner et al. conducted a retrospective review of 100 consecutive patients who had a revision TKA. Some of the patient's received either a closed suction or reinfusion type drain. Fifty-two percent of the patients were female, which was lower than the 75% in our cohort. Fifty-eight percent of their patients participated in the preoperative autologous donation program (at least one unit of blood), and a significant decrease in preoperative Hgb levels was found. They noted that the preoperative autologous donation resulted in an “orthopaedic-induced anemia.” They also concluded that autologous donation may not be appropriate because it predisposed patients to transfusion. All control patients included in our study had mild anemia (10–13 g/dL) prior to surgery (whether preoperative autologous donation was utilized or not). Greater than half of our control patient's were transfused (17% allogeneic) with an average of 256cc's of blood.

De Andrade et al. compared epoetin-*α* to a placebo in a primary knee arthroplasty double-blind study. They found that those with a mild anemia (10–13 g/dL) who received epoetin-*α* (300 IU/kg or 100 IU/kg) had a lower allogeneic transfusion rate relative to placebo [21]. Additionally, Stowell et al. found that epoetin-*α* weekly doses of 40,000 units raised hemoglobin levels from 12.3 g/dL to 13.8 g/dL, preoperatively. Their patients maintained higher levels peri- and postoperatively compared to a cohort who had preoperative autologous donation [22]. Our revision patients responded with an increased hemoglobin level from pre- to postop of 11.97 g/dL to 13.93 g/dL, respectively. We believe this change was clinically significant and most likely prevented peri- or postoperative transfusions, decreased length of stay, and allowed patients to more actively participate in physical therapy sooner [20, 21].

According to Sehat et al., there may be hidden blood loss into the soft tissue and joint of an arthroplasty patient [14]. In addition, two studies using radiolabeled RBC's showed unexplained peri-operative blood loss likely into tissue compartments [23, 24]. Our study was not powered to detect such a difference in hidden and total blood loss between patient groups; however, our average total and hidden blood losses were elevated when compared to the primary knee patient [14]. In addition, when we attempted to evaluate total and hidden losses, we noted a few important imperfections. For example, the estimated blood loss (EBL) is a nonstandardized subjective measure that likely varies as much inter- as intrainstitutionally. Moreover, the median value has often been used to report hidden and total blood loss findings because the range of losses had tremendous variability [13]. However, an interesting finding derived from such calculations is that a total knee arthroplasty, on average, has a higher hidden blood loss than total hip arthroplasty [14]. The change in hemoglobin from pre- to postop in our study cohort was 3.24 g/dL. Such a change was similar to those recorded by Sehat et al. who noted that a TKA without reinfusion had a change of 3.3 g/dL and 2.8 g/dL after re-infusion [14]. 

Our study resulted in not only an increase in preoperative hemoglobin levels, but also a higher hemoglobin level at time of discharge. This is substantial considering the control patients discharge counts included blood transfused. 

We performed all cases under tourniquet control, which is thought to cause fibrinolysis [25, 26]. Furthermore, postoperative fibrinolysis is also thought to occur transiently [27]. This increased activity may elevate blood loss after revision total knee arthroplasty [28, 29]. Interestingly, epoetin-*α* has been found to transiently increase the number of circulating platelets as well as improve their function. This may potentially decrease total blood loss for the revision knee patient [30]. There is also an antiapoptotic activity that in preclinical and small clinical studies has been shown to protect cells from hypoxic and ischemic events [31–33]. However, cancer and chronic renal failure trial patients had an increased risk of thrombotic complications and death [34–36]. Our study population developed one uncomplicated deep venous thrombosis (3.6%). However, one control patient developed a pulmonary embolism (2.3%). The control patient was a 93 yo female with an ASA score of 3 and BMI of 31, while the study patient was a 63 yo male with an ASA score of 4 and BMI of 31; neither patient had a history of deep venous thrombosis or pulmonary embolus in their past. All patients received the same postoperative treatment course that consisted of antithromboprophylaxis, early ambulation, and physical therapy. Additionally, no study patient had an ischemic event. 

No study is without limitation. First, we collected all data retrospectively and there are inherent limitations in such study design. Therefore, we attempted to reduce potential confounding by patient-matching based on age, gender, BMI, and American Society of Anesthesiology (ASA) scores. We also included a consecutive series of case patients. Second, spinal anesthesia is known to be associated with less perioperative blood loss in TKA when compared to general anesthesia [37]. The majority of our patients underwent spinal anesthesia but a few patients required general anesthesia. Third, the percentage of patients who received pre-op iron, folic acid, vitamin B12, and multivitamins was elevated in the study group which may have enhanced the effects of our epoetin-*α* dosing regimen. Fourth, our patient selection was limited to those without a thromboembolic history. Fifth, the triggers for transfusion are physician dependent. To better control for this, we only included cases performed by two surgeons who utilize similar transfusion protocols. Furthermore, those patients treated with epoetin-*α* did not require any transfusions. Therefore it is unlikely that a true clinical difference in transfusion criteria occurred between our cohorts. 

In conclusion, the present study may suggest that epoetin-*α* has a role in reducing the need for blood transfusion in the mildly anemic patient who undergoes revision knee surgery. It may also decrease patient length of stay allowing for earlier readiness to resume normal activities or meet short-term milestones [20]. Its use may also be an attractive alternative to autologous donation. A randomized study to evaluate the direct and indirect costs of such a treatment methodology in the mildly anemic revision patient may be warranted.

## Figures and Tables

**Table 1:** Demographic data.

Characteristic	Epoetin-*α*	Control	*P*-value
Age (y)	63.7	64.4	0.707
BMI (kg/m^2^)	35.9	32.5	0.125
ASA score (no.)	2.59	2.48	0.663
INR	1.056	1.06	0.427
Platelet count (per mm^3^)	263,070	237,360	0.761
Sg Duration (min)	85.4	92.7	0.276
Length of Stay (days)	3	3.67	0.042

**Table 2:** Blood data.

Characteristic	Epoetin-*α*	Control	*P*-value
Average epoetin-*α* dose 40,000 u	2.86	0	Na
Epoetin-*α* effect on Hb (g/dL)	11.97 to 13.8	Na	<0.001
Hb at Sg (g/dL)	13.8	12.2	<0.001
Hb on discharge (g/dL)	10.6	9.69	<0.001
Transfusion (%)	0	54	<0.001
Mean TBL (mL)	1670	1737	0.730
Mean HBL (mL)	1578	1643	0.750
Median TBL (mL)	1555	1778	Na
Median HBL (mL)	1397	1682	Na
CBL (mL)	1401	1668	0.140
Mean EBL (mL)	104.2	120.6	0.618

**Table 3:** Component revisions.

Characteristic	Epoetin-*α*	Controls
Component Revisions	Number (%)	Number (%)
Tibia and femur	23 (82.1%)	43 (84.3%) 23 transfused
Femur	1 (3.6%)	1 (1.75%) 1 transfused
Tibia	1 (3.6%)	2 (3.5%) 1 transfused
Patella	1 (3.6%)	3 (5.3%) 1 transfused
Polyethylene exchange	2 (7.1%)	4 (7.01%) 2 transfused
